# New frontiers for platelet CD154

**DOI:** 10.1186/s40164-015-0001-6

**Published:** 2015-03-01

**Authors:** Antoine Dewitte, Annabelle Tanga, Julien Villeneuve, Sébastien Lepreux, Alexandre Ouattara, Alexis Desmoulière, Christian Combe, Jean Ripoche

**Affiliations:** INSERM U1026, and Université de Bordeaux, F-33000 Bordeaux, France; Cell and Developmental Biology Programme, Centre for Genomic Regulation, 08003 Barcelona, Spain; Department of Molecular and Cell Biology, Howard Hughes Medical Institute, University of California, Berkeley, CA 94720-3200 USA; Service d’Anesthésie-Réanimation II, CHU de Bordeaux, F-33600 Pessac, France; EA 6309, University of Limoges, F-87025 Limoges, France; Service de Néphrologie Transplantation Dialyse, CHU de Bordeaux, F-33076 Bordeaux, France

**Keywords:** Platelets, CD154

## Abstract

The role of platelets extends beyond hemostasis. The pivotal role of platelets in inflammation has shed new light on the natural history of conditions associated with acute or chronic inflammation. Beyond the preservation of vascular integrity, platelets are essential to tissue homeostasis and platelet-derived products are already used in the clinics. Unanticipated was the role of platelets in the adaptative immune response, allowing a renewed conceptual approach of auto-immune diseases. Platelets are also important players in cancer growth and dissemination. Platelets fulfill most of their functions through the expression of still incompletely characterized membrane-bound or soluble mediators. Among them, CD154 holds a peculiar position, as platelets represent a major source of CD154 and as CD154 contributes to most of these new platelet attributes. Here, we provide an overview of some of the new frontiers that the study of platelet CD154 is opening, in inflammation, tissue homeostasis, immune response, hematopoiesis and cancer.

## Introduction

**Figure 1:**
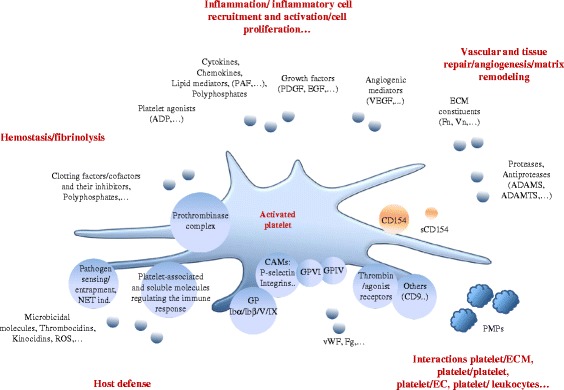
**Platelets have a pleiotropic range of biological roles that extend beyond hemostasis.** The breaching of tissue homeostasis leads to platelet activation, a common event in various causes of tissue injury, traumatic, infectious, ischemic, autoimmune… Platelet activation, apart from its essential role in bleeding arrest, is the source of a flow of information that fuels the inflammatory reaction. Platelets represent host defense machines against infection, via the clearing of pathogens and the expression of membrane-bound and soluble signals that regulate the innate and adaptative arms of the immune response. Pathways activated in inflammation, coagulation, vascular/tissue repair and host defense are connected via soluble and cell-mediated signals, providing a coherent biological response aiming at arresting bleeding, curing infection and reestablishing tissue homeostasis. CD154 interfaces with many of these pathways (see Figures 2 and 3); activated platelets express a membrane-bound form of CD154 and release a soluble form (sCD154). Platelet derived microparticles (PMPs) recapitulate several of activated platelet functions (see text for details). Only some relevant molecules have been depicted. Small circles symbolize secreted molecules, large circles membrane-associated molecules. Abbreviations: CAMs, cell adhesion molecules; Fg, fibrinogen; Fn, fibronectin; ECM, extracellular matrix; NET ind., neutrophil extracellular traps induction; PAF, platelet activating factor; ROS, reactive oxygen species; Vn, vitronectin; vWF, von Willebrand factor.

Platelets are cytoplasmic fragments released in the bloodstream during the fragmentation of polyploid megakaryocytes (MK), a phenomenon critically dependent on thrombopoietin [1-3]. The mammalian platelet is thought to result from a phylogenic trend to ensure hemostasis under high vascular shear forces; indeed, it can specifically form arterial thrombi sustaining high shear stress [4]. It is thought that the platelet coopted attributes of a nucleated cell ancestor endowed with a multifunctional role in coagulation, inflammation and defense against infections [5,6]. Platelets have a short lifespan, of around 7 days; mechanisms responsible for their clearance are ill-understood; lectin-carbohydrate recognition of aged and damaged platelets by splenic and liver macrophages and hepatocytes is emphasized [7]. The best-defined function of platelets is hemostasis. Disruption of the endothelial cell (EC) lining leads to platelet activation, platelet adherence and aggregation which temporarily plug the damaged vessel. In this process, platelets also drive and confine coagulation at sites of tissue damage. Indeed, deficiencies in platelet production or function are associated to bleeding disorders, while increases in platelet number or gain of function are associated to thrombosis. The role of platelets in health and disease extends beyond hemostasis; non-hemostatic platelet functions include inflammation, innate and adaptative immune responses and tissue homeostasis (Figure 1). Decisive advances in understanding platelet function have been made through the characterization of platelet receptors and their ligands and platelet-derived mediators [8]. Among platelet mediators, CD154, the ligand of CD40, has attracted specific attention as it orchestrates many of these new platelet attributes.

### CD154

CD154, the CD40 ligand, a member of the Tumor Necrosis Factor (TNF) family, is central to the immune response [9,10]. CD154 was discovered as mediating humoral immunity and was originally considered to be restricted to activated helper T cells. The CD154/CD40 interaction drives B cell proliferation, antibody production and isotype switching and is involved in thymic selection. This interaction is required for B memory cell generation and germinal center formation. Accordingly, CD154 deficiency is associated with an impairment of the humoral immune response to T-cell dependent antigens, including defective immunoglobulin class switching; patients with the X-linked hyper-IgM syndrome caused by mutations of the *CD154* gene, generally present low serum IgG and IgA, but normal or increased serum IgM, and are susceptible to opportunistic infections. Mice with a disrupted *Cd154* gene fail to undergo isotype switching to T-cell dependent antigens while normally responding to T-cell independent antigens. In line with its regulatory role on the adaptative immune response, the CD40/CD154 interaction contributes to autoimmune disorders in a number of animal models [11-15]. Manipulation of the CD154/CD40 interaction has been used in efforts to develop novel strategies in autoimmune diseases, results in animal models being encouraging [13]. Clinical trials have been launched with humanized anti-CD154 monoclonal antibodies. Clinical interest of this strategy remains mixed, and is strongly limited by thrombotic complications [12-14].

Apart from B cells, CD40 is expressed by various cells, including dendritic cells (DC), monocytes, T lymphocytes, EC, a variety of epithelial cells, smooth muscle cells, fibroblasts; its expression is low in basal conditions and is stimulated by inflammatory mediators [16-19]. CD40 expression is increased by CD154, however it is not known whether this induction is direct or indirect [20,21]. CD40 is not the sole receptor for CD154; alternative receptors have been described, such as integrins α5β1, αIIbβ3 and αMβ2; CD154 binding depends on their activation states [22-25]. These additional receptors are of significance in the pathophysiology of atherogenesis and are important to consider when comparing CD40- and CD154-deficient mouse phenotypes.

CD154 is a transmembrane protein and a proteolytic soluble form, sCD154, which keeps the CD40-binding domain, is released by a partially understood mechanism. The release of sCD154 was first documented in activated T-lymphocytes [26]. CD154 has a trimeric configuration, required for functional activity [27-30]. A complex signaling cascade is triggered by CD40 ligation, involving TNF receptor-associated factors (TRAF) as proximal transducing signal initiators [10,20]. Several signaling pathways, including nuclear factor-κB (NF-κB), c-Jun N-terminal kinase (JNK) and p38 mitogen-activated protein kinase pathways, are activated by CD40 ligation; however, there is a differential outcome depending upon which TRAF member binds preferentially, and which cell/conditions are involved [31]; the binding of TRAF-6 is critical in vascular inflammation and metabolic complications associated with obesity [32,33].

CD154 expression is also observed in natural killer cells, DC, cells of the monocyte/macrophage lineage, endothelial, smooth muscle and epithelial cells [20]. Basal CD154 expression is very low, or undetectable, as in EC and epithelial cells for example [34], and is increased by a variety of stimuli, most notably inflammatory cytokines [20]. This suggests that CD154 expression may mostly have relevance when induced, as in inflammation. CD154 is also expressed by blood platelets, being cryptic in unstimulated platelets and rapidly exposed at the platelet surface following platelet activation [35].

#### CD154 expression by platelets

The distribution of CD154 in platelets is partly understood. CD154 was found in α-granules, as shown by immunoelectron microscopy or quantitative immunofluorescence approaches [36,37]. Accordingly, patients presenting a Gray-platelet syndrome, are characterized by platelets that lack α-granules, and do not release CD154 upon activation [37]. CD154 is highly coclustered with insulin growth factor in α-granules, the signification of which is unknown [36]. One question is whether CD154 is also cytosolic, as found in resting platelets [38].

Pre-mRNAs and mature mRNAs are present in platelets and a functional spliceosome and translational apparatus allow platelets to process them, in response to platelet-activating signals [39,40]. Detecting CD154 mRNA by RT-PCR in platelets is challenging because of purity issues. However, CD154 mRNA was evidenced in mouse platelets, introducing other potential regulatory layers of CD154 expression by platelets [34].

#### When activated, platelets express a membrane form and release a soluble form of CD154

Platelets are activated by immobilized or soluble agonists. The activation-driven secretion of granule content is a primary phenomenon [41-46]. Platelets also synthetize mediators, including interleukin-1β, tissue factor (TF), fibrinogen, thrombospondin, von Willebrand Factor, αIIbβ3, through a translational-dependent pathway triggered by platelet activation [47,48].

Soluble CD154 is released by an activation-driven proteolytic mechanism. Agonists, including thrombin, thrombin receptor-agonist peptide, ADP or collagen, stimulate CD154 expression at the platelet membrane and the release of sCD154; long-term platelet activation leads to complete conversion of CD154 to sCD154 [38,49-53]. A matrix metalloproteinase (MMP)-dependent proteolytic event is involved. The involvement of MMPs, MMP-2 and/or MMP-9, [51,54-57], differs from the release of sCD154 by activated T-cells, which involves ADAM10 and 17 [58]. A role for αIIb/β3 has been put forward, as αIIb/β3 antagonists inhibit sCD154 release and as Glanzmann platelets show reduced sCD154 release rate [53,54,59]. An interaction between αIIb/β3 and MMP-2 is involved [57]. The roles of NADPH activation and reactive oxygen species (ROS) generation as well as CD154 binding to platelet CD40 have been underlined [50,60]. The particularity of sCD154 release may explain its specific response to agonists and secretion kinetics [38,53]; however, how sCD154 is released remains be fully understood, as shown for example by the effects of inhibitors added after platelet activation, suggesting complex, intra-platelet mechanisms [53]. A debate remains about the parallel biological activities of platelet-derived soluble and membrane-associated CD154; recombinant soluble forms, particularly trimeric forms, are active [50,61-63]. Finally, sCD154 activates platelets by itself, suggesting feed-back amplification of its secretion [64,65].

#### The megakaryocytic origin of platelet CD154

The assembly and loading of granules mainly occur in MK; granules are distributed in proplatelets via a microtubule-dependent mechanism [2,66,67]. The main origin of platelet CD154 is likely to be the MK that express CD154 mRNA, as shown in MK derived by differentiation of human and mouse hematopoietic progenitor cells and in MK of immune thrombocytopenic purpura (ITP) patients [68,69]. CD154 mRNA expression is increased upon MK differentiation [69]. CD154 protein is also found in MK cell lines and in MK from ITP patients [38,68,69]. As for T cells, the calcium-dependent activation of nuclear factor of activated T cells-c2 and the early growth response transcription factor EGR-1 contribute to *CD154* gene activation in MK [69,70].

Translation from endogenous mRNAs contributes to platelet content. Its significance in quiescent platelets is unclear. However, pre-mRNA processing and mRNA translation are driven by platelet activation [40,48,71]. The contribution of such mechanism in CD154 expression during platelet lifespan is unknown.

Platelets also carry mediators present in plasma and possibly concentrated and/or modified within platelets [72,73]. Fibrinogen, albumin, immunoglobulins, amino acids, inflammatory and angiogenic mediators including vascular endothelial growth factor (VEGF), histamine or serotonin, are among them. Soluble CD154 is not detected in platelets, making unlikely its uptake from plasma.

#### Platelets are a significant reservoir of CD154 in the organism

Platelets carry approximately 5 ng of CD154/mL of blood [52]. Correlation studies suggest a link between platelet count and plasma or serum sCD154 [37,52,74-78]. Such a correlation is also found in experimental ITP [78]. In ITP, albeit platelet CD154 is elevated [68], plasma sCD154 is reduced [78], again suggesting relationship between the platelet count and circulating sCD154. However, there are contrasting studies, and a correlation between the platelet count and sCD154 is not always found [79,80].

Importantly, platelet activation is associated to elevated sCD154 and, indeed, platelet activation markers correlate with sCD154 in blood [81-83]. For this reason, serum seems inappropriate to evaluate circulating sCD154; in fact, sCD154 levels are higher in serum than in plasma, clotting resulting in increased sCD154 generation [52,79,80,84-88]. Hence the importance of a preanalytical standardization of blood samples processing, conditions such as temperature, length of storage, centrifugation, interfering with measurement [84,89]. Further, plasma/serum sCD154 may correspond to a pool of free soluble and microparticle-bound CD154 [84] and ELISA may not discriminate between sCD154 and platelet microparticles (PMP)-associated CD154 [90]. Circulating sCD154 is linked to platelet activation state; in patients with recent thrombotic events, plasma sCD154 correlates with platelet count, but this correlation is not found in patients with non-thrombotic, non-inflammatory conditions [84]. Finally, in patients with cardiovascular conditions, commonly used drugs such as statins, interfere with sCD154 releasing, a point that has also to be considered [91-93]. The baseline presence of sCD154 in the plasma of healthy subjects may be secondary to basal platelet activation, as in high shear stress flow areas [94]. PMP are released upon platelet activation [95]. A functional CD154 is expressed by PMP [63,96]. The importance of the contribution of PMP-bound CD154, in comparison with the “true” soluble CD154, to plasma sCD154 has been emphasized [90]. Questions also remain on the fate and half-life of sCD154 in blood and how the CD154 information can be delivered at distance from platelet activation sites.

#### Platelet CD154: a critical mediator of the inflammatory reaction

**Figure 2:**
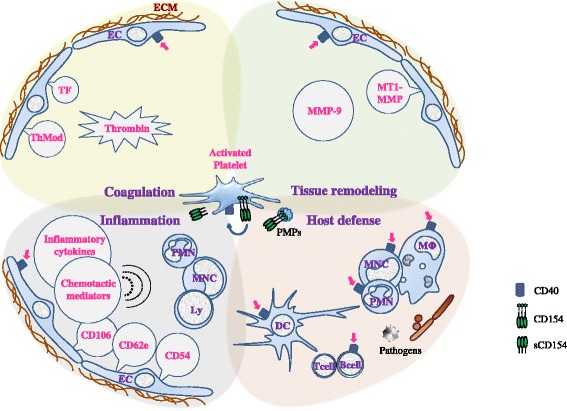
**CD154 is a universal contributor to platelet functions.** Activated platelets display CD154 at their membrane and release a soluble form (sCD154). Platelet CD154, directly or indirectly, is a molecular driver of inflammation, coagulation, tissue remodeling, and host defense, processes that intersect at multiple levels. Endothelium is a primary target. Platelet CD154 induces tissue factor (TF) expression and activity, thereby contributing to thrombin generation and upregulates urokinase plasminogen activator receptor which is at the interface between fibrinolysis/inflammation/tissue remodeling. MMP-9 and MT1-MMP induction contribute to regulate endothelium proteolytic activity [97]. Platelet CD154 also induces pro-inflammatory and chemotactic (dotted semicircles) molecule expression, and adhesion molecule expression (CD62e, CD54, CD106,…), leading to leukocyte recruitment and activation. Once activated, target cells recruit and activate other cells through multiple inputs; several amplification loops are thus generated including platelet activation by sCD154 itself (blue arrow). Platelet CD154 also activates cellular effectors of the innate and adaptative immune responses, polymorphonuclear cells (PMN), monocytes (MNC), macrophages (MΦ), dendritic cells (DC); how platelet CD154 contributes to host defense is schematized in Figure 3. CD154-expressing platelet microparticles (PMPs) share most of these functions. Depicted molecules do not comprehensively represent the range of platelet-derived mediators that are controlled by platelet CD154, and other interfaces, such as with endothelin-1, continue to be identified. Magenta arrows depict interaction with CD40. Dotted line for thrombomodulin (ThMod) represents inhibition; full line for others represents stimulation. Dotted semicircles symbolize chemotaxis. Abbreviations: Ly, lymphocytes; MMP, matrix metalloproteinases.

Platelets orchestrate a subtle balance between tissue injury and repair; they are a key source of material for reestablishing tissue homeostasis but they also contribute to tissue injury. CD154 mediates several platelet functions in tissue homeostasis (Figure 2).

##### Platelet CD154 and inflammation

Regardless of its cause, the inflammatory milieu is rich in platelet-activating material, including chemokines [98]. The dialog between EC and platelets in inflammation has been widely studied as EC are primary platelet partners. Upon CD40 ligation, EC switch to an activated phenotype, expressing molecules that contribute to an inflammatory and thrombotic scenario, including cytokines/chemokines, adhesion molecules, and tissue factor [16,20,99]. Platelets/EC reciprocal activation is critical in atherosclerosis and cardiovascular conditions [100-103]. The pathogenic role of platelet CD154 is a major theme in atherosclerosis and cardiovascular diseases [25,62,74,100-109].

The role of platelet CD154 in inflammation extends beyond the dialog with EC, as activated platelets interact with various CD40 expressing-cells. Platelets are brought to inflammatory sites via vascular injury/permeability, attachment to activated leukocytes, and also chemotactic recruitment [110]. CD40 ligation on inflammatory cells at sites of tissue injury is a potent stimulus for the expression of a variety of proinflammatory mediators including cytokines, chemokines, eicosanoids, products of the proteolytic cascades, ROS generation, and of adhesion molecules [49,111], making platelet CD154 a versatile fuel for inflammation. The platelet contribution in many inflammation-associated disorders, including rheumatic, lung, gastrointestinal, neuro-inflammatory and metabolic diseases is actively studied [112-120] and the specific pathogenic role played by platelet CD154 in these disorders is a recently opened frontier. Soluble CD154 levels were found to correlate with disease activity as in systemic lupus erythematosus [121]; whether sCD154 could represent a potential useful marker in inflammation-associated disorders is an interesting question. PMP also contribute to inflammatory disorders [122-128]; the specific role of PMP-associated CD154 remains however to be fully understood.

##### Platelet CD154 and tissue repair

The effectors of inflammation are orchestrated to cure infection and restore tissue integrity [129-131]. At various steps of tissue repair, platelets are a source of relevant material, including growth factors, pro- and anti-apoptotic mediators, matrix and matrix remodeling proteins [132-135] (Figure 1). Platelets contribute to maintain resting and injured endothelium integrity [136]. On injured endothelium, platelets provide EC growth-promoting and anti-apoptotic mediators, attractants for progenitor cells endowed with vascular healing properties [135]. They contribute to restoring the vascular network, by secreting regulators of angiogenesis [137-139]. Beyond endothelium, a remarkable role for platelets in organ regeneration has been substantiated. Platelets contribute to liver regeneration, serotonin being essential [140-142]. It is tempting to speculate that platelets will be found to have a broader role in organ regeneration by providing key mitogenic signals in various organs, such as for example fibroblast growth factor or platelet-derived growth factor that contribute to muscle or brain repair [143,144]. This is also in line with the known ability of platelet lysates to sustain the growth of primary cell cultures. PMP also contribute to vascular integrity [145-148] and promote tissue repair [128,149]. Platelet products have already found various applications in the clinics [150-154].

The specific role of CD154 has been mainly studied in EC. CD154 promotes EC survival, proliferation and migration, capillary-like tube formation *in vitro* and angiogenesis *in vivo*. Mechanisms include activation of the phosphatidylinositol-3 kinase/Akt pathway, induction of angiogenic mediators and matrix remodeling protein production [155-157]. CD40 signaling contributes to neointima repair, TRAF6 signaling intermediate being critical [32,158,159]. However, platelet CD154 was shown to inhibit the VEGF-induced EC migration via increased ROS generation, and sCD154 to inhibit VEGF-induced angiogenesis [160]. Soluble CD154 also promotes oxidative stress in endothelial outgrowth cells (EOC), reducing their viability and proliferation [161], while promoting endothelial repair via increased production of MMP-9 by EOC [162]. These findings may be context-dependent; they emphasize the importance of platelet CD154 in vascular homeostasis and the complexity of its biological interfaces. Other tissues for which platelet CD154 is likely to show importance for repair are skin and bone. CD40 ligation stimulates keratinocyte differentiation, suggesting contribution to skin wound repair [163]. Regulation of osteoclastogenesis by CD154 is suggested by the reduced bone mineral density together with elevated urine markers of osteoclast activity in patients with the X-linked hyper-IgM syndrome, and the reduced bone mineral density in CD154 deficient mice [164,165]. CD40 is expressed by osteoblastic cells and CD154 is anti-apoptotic in these cells [166]. Therefore, much remains to be found about the role of platelet CD154 in tissue repair. As CD40 is largely distributed, platelet CD154 could be conjectured to be generally involved, to one degree or another, in tissue repair.

##### Platelet CD154 as a mediator of tissue injury

The model of platelets promoting tissue repair is to be compared to their deleterious role in acute and chronic tissue injury. Difficult points are raised by this friend or foe facet, implicating balanced therapeutic approaches [119]. Ischemia/reperfusion (I/R) underscores platelet deleterious role, and the importance to control platelet activation in this context. In I/R, platelet activation in the microcirculation vascular bed leads to tissue injury, as shown in lung, liver or kidney. Platelet depletion or antiplatelet treatments are protective in several experimental I/R models [167-169]; CD154 is contributing: mice deficient in CD154 are protected from I/R-mediated injury in brain, lung, liver or intestine; in lung I/R-mediated injury platelet CD154 is specifically contributing [170-172].

#### Platelet CD154 and the immune response: unanticipated new frontiers

**Figure 3:**
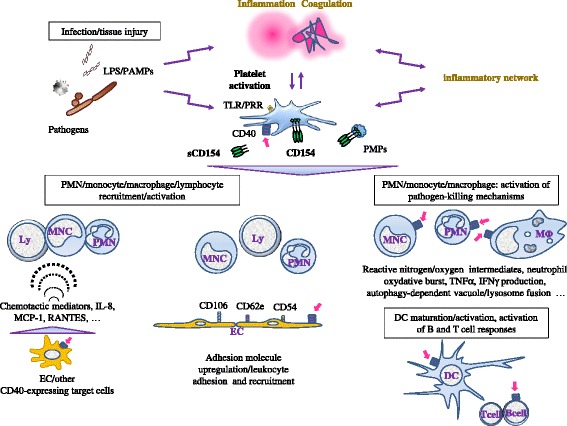
**Platelet CD154 contributes to the host defense against infections.** Infection triggers inflammation and coagulation. The interaction with pathogens, pathogen-derived molecules such as lipopolysaccharide (LPS), inflammation and coagulation concur to activate platelets, leading to CD154 display at the platelet membrane and the release of soluble CD154 (sCD154). Multiple inputs amplify the platelet activation scenario, including soluble and cellular effectors of the inflammatory network. Platelet CD154 targets several immune response effectors, including contribution to the chemotactic recruitment (dotted semicircles symbolize chemotaxis) of leukocytes to sites of infection, *e.g.* through the induction of adhesion molecules on EC (CD62e, CD54, CD106) and activation/upregulation of integrins such as αMβ2 on neutrophils [158,200]. CD40 triggering is a major inducer of pathogen-killing mechanisms by phagocytic cells. These responses are amplified by inflammatory mediators generated upon CD40 ligation; this schematic representation does not represent all interfaces that are directly or indirectly regulated by platelet CD154. Platelet CD154 influences the adaptative immune response, through several mechanisms, including the activation/maturation of antigen presenting cells (see text for details). Magenta arrows depict interaction with CD40. Abbreviations: PAMPs, pathogen-associated molecular patterns; PRR, pathogen recognition receptors; TLR, Toll-like receptors.

Platelets participate to the control of infection via direct and indirect mechanisms [6,173-178]. The significance of platelet Toll-like receptors (TLR) has been emphasized; TLR ligation activates platelet secretion of mediators regulating the immune response, including sCD154 [6,179-184]. Platelets also regulate several steps of the adaptative immune response [6,182-194]. Moreover, platelets can present antigen [195]; they express MHC class I molecules and T cell costimulatory molecules, including CD86 and CD40 and harbor a functional proteasome [196-199]. Among platelet mediators, CD154 proved to be critical in linking platelet and immunity (Figure 3).

Although much remains to be understood, particularly with reference to the innate immune response, the specific role of platelet CD154 in immunity is strengthening. Several pathogen-clearing mechanisms are stimulated by CD154, including platelet aggregation [173], phagocytosis and production of defense proteins, such as complement proteins and interferon-α, by cells of the innate immune system [6,20,201]. CD40 contributes to the regulation of innate immune response, including induction of TLR expression, cooperation in TLR-mediated B cell activation, engagement in the crosstalk between intracellular MHC class II molecules and TLR signaling pathway [202-204]. The specific role of platelet CD154 in these mechanisms remains to be precised. However, it is now appreciated that platelet CD154 controls many facets of the interface between innate and adaptive immune responses [173,187,191,205]. Platelet CD154 induces DC maturation, can activate B cells, antibody production and isotype switching, contributes to germinal center formation, and enhances CD8^+^ T cell responses [188,206-213]. Platelet CD154 helps mounting a protective cytotoxic T cell immune response to viral or bacterial challenge [206,214]. Platelet CD154 may promote the immune response in the context of low antigen challenge by lowering the antigen threshold, and improve B cell response in regulatory T-cell limiting settings [210,215]. Further, sCD154 *per se* induces cardiac allograft rejection [212]. Many questions remain. How platelet CD154 enters the draining lymph nodes to regulate the adaptive immune response machinery is not known; PMP may convey this information, as CD154 associated to PMP is functional: it enhances DC activation, germinal center formation, B cell proliferation and IgG production [63,216]. Several questions are also raised with reference to platelet CD154 in autoimmunity; this “dark side” [14,217] feature of platelet CD154 is a recently opened frontier. Platelet CD154 is competent to increase production of antiplatelet antibodies in immune thrombocytopenic purpura [68] and, in systemic lupus erythematosus, platelet CD154 activates antigen presenting cells contributing to enhanced interferon-α production [218].

#### Platelet CD154: a new hematopoietic regulator?

Hematopoiesis can be adapted in response to inflammation/infection by signals generated at bone marrow distal sites [219-224]. Platelets are activated at sites of inflammation/infection and are a major source of circulating sCD154. Could platelets deliver a CD154 signal, through sCD154, platelet- or PMP-associated CD154 that regulates hematopoiesis? Platelet mediators enhance hematopoietic stem cell proliferation and platelet-derived signals may contribute to CD34+ cell mobilization [225,226]. Several studies have demonstrated CD154 involvement in hematopoiesis. CD154 regulation of early B cell lymphopoiesis is suggested by the sCD154-induced increased number of B cell progenitors (BCP) in mice after bone marrow transplantation (BMT) [227]. CD40 is expressed on BCP, and a positive effect of CD40 ligation on BCP proliferation can be observed on pre- and immature B cells in human and pro-B cells in the mouse [228,229]. In the mouse, there is clear experimental evidence for a positive role of CD154 in B cell hematopoiesis and, particularly in stress conditions, as after BMT [229]. However, normal numbers of circulating B cells in patients with X-linked hyper-IgM syndrome would rule out an absolute requirement for the CD154/CD40 signaling in early B cell development. CD154 may therefore mostly play a significant role in emergency B cell hematopoiesis [229]. More is known about CD154 regulation of the lymphoid system maturation, which has been fully reviewed [230]. A role for platelet CD154 on myelopoiesis is suggested by the sCD154-mediated increased granulocyte and platelet recovery after BMT in the mouse and by the neutropenia and thrombocytopenia observed in patients with X-linked hyper-IgM syndrome [227]. *In vitro*, sCD154 promotes the differentiation of CD34+ cells towards the granulocytic/monocytic and megakaryocytic lineages in CD34+/stromal cell cocultures. The mode of action of sCD154 appears to be essentially indirect, through the induction of hematopoietic cytokines by bone marrow stromal cells [231,232]. Platelet CD154 may therefore play a role in regulating emergency hematopoiesis. However, many questions remain unsolved, particularly which and how platelet CD154 signals could be delivered and interact with bone marrow stem/progenitor cells.

#### Platelet CD154 and cancer: a rapidly expanding frontier

There is strong evidence for the involvement of platelets in cancer progression; mechanisms are multiple [233-240]. Platelets are activated in the tumor environment and bind tumor cells. Mediators released upon platelet activation are key to tumor angiogenesis [241,242] and are likely to contribute to the tumor-supporting inflammatory environment [243,244]. Platelets play a positive role in metastasis [234,238,245-249]. However, this may not be true for all organs [250]. In hematogenous dissemination, platelet/cancer cell microthrombi provide protection, including shielding from shear flow, or immune evasion; during the arrest and extravasation phases, platelet mediators facilitate tumor cell arrest on EC, extravasation, survival and growth after seeding [251]. Platelet MPs are also contributing [124,252,253].

Many tumor cells express CD40. The outcomes of CD40 ligation on tumor cells are ambivalent depending on the models studied. In one hand, CD40 ligation promotes anti-tumor immune surveillance through a variety of mechanisms including antigen-presenting cell activation, restoration of malignant cell immune recognition, activation of tumoricidal-infiltrating macrophages, immunostimulatory cytokine production. CD40 ligation also induces tumor growth arrest and sensitization to apoptotic signals. On the other hand, CD40 ligation has positive consequences on tumor growth, survival and resistance to chemotherapy and metastatic potential. The interpretation of CD154 effects on cancer cells is made complex, first by the existence of several receptors for CD154, potentially explaining variable outcomes of CD154 treatment of tumor cells, and second, by the difficulty in assessing direct versus indirect effects. The contribution of the CD40 signaling in cancer, and prospects offered by targeting the CD40 signaling for cancer treatment have recently been underlined and reviewed [254-258]. However, the specific role played by platelet CD154 remains a new important frontier. If platelet activation is likely to result in expression of CD154 and generation of sCD154 in the tumor cell environment, this study is made complex as there are extra platelet sources of CD154.

## Conclusion

There have been recent and rapid advances in our current knowledge of the non-hemostatic functions of platelets, placing them in the middle of the spectrum of mechanisms that maintain homeostasis, and highlighting their role in a variety of inflammatory and immune disorders. However, platelets store and release such a wide diversity of biologically active mediators that major gaps remain in our understanding of which and how these mediators collectively fulfill these functions. Platelet CD154 has attracted considerable attention as it recapitulates several of non-hemostatic platelet attributes. Considering the large number of different cells expressing CD40, the complex signaling cascade and the wide range of effectors activated by the CD154/CD40 interaction, it can be anticipated that future investigations will further extend the contribution of platelet CD154 in health and disease. For example, recent publications on the CD154/CD40 dyad have pointed to its role in obesity and hepatic steatosis [259-263], and it is tempting to speculate that platelet CD154 contributes to metabolic homeostasis. In the same direction, the number of physiological or pathological conditions associated with platelet activation is enlarging. For example, platelet activation has been found associated to aging, to emotional or environmental stresses…; platelet CD154 might represent a significant link between these conditions and accompanying pathologies, such as cardiovascular events [264]. However, platelet CD154 is always acting in a multicytokine context, including inhibitors and activators released at the same time by platelets; understanding how this complexity is tuned and evidencing the specific role of platelet CD154 remains a difficult challenge.
